# Thymoma With Myasthenia Gravis: A Study of Two Cases

**DOI:** 10.7759/cureus.67739

**Published:** 2024-08-25

**Authors:** Aravindan Kumaravel, Sulochana Sonti

**Affiliations:** 1 Department of Pathology, Saveetha Medical College and Hospital, Saveetha Institute of Medical and Technical Sciences (SIMATS) Saveetha University, Chennai, IND

**Keywords:** thymoma-associated myasthenia gravis, prognosis, pathogenesis, histopathology, thymoma

## Abstract

Thymoma is a rare, primary neoplasm of the thymus gland, commonly presenting in adults and associated with autoimmune diseases, most commonly myasthenia gravis (MG). Despite its generally indolent behavior, the variability in clinical presentation and potential for malignancy necessitates detailed evaluation and management. In this report, we present two cases: a 41-year-old male and a 39-year-old female, both of whom presented with a mediastinal mass with symptoms of myasthenia. Further investigation, including imaging and histopathological examination, confirmed the diagnosis of a type B2 thymoma and type B1 thymoma for the male and female patients, respectively. The patients underwent successful complete surgical resection of the masses, with the postoperative recovery being uneventful. They were monitored for signs of disease recurrence and associated autoimmune conditions during follow-up visits. This report underscores the importance of early detection and thorough clinical evaluation of thymoma, particularly in patients with associated paraneoplastic syndromes. Complete surgical resection remains the cornerstone of treatment, with adjuvant therapy tailored based on individual risk factors. Ongoing surveillance is crucial for identifying potential recurrences and associated conditions.

## Introduction

Thymoma is a benign neoplasm of the thymus, which usually presents in adult life, with a peak incidence in the fifth and sixth decades [1]. It presents mainly in the anterosuperior mediastinum [2]. Their clinical significance is underscored by associations with various autoimmune diseases, particularly myasthenia gravis (MG), which occurs in approximately 30-50% of patients with thymoma. The cell of origin is the epithelial cells of the thymus gland, and thymoma has an incidence of 0.13-0.26 cases per 100,000 population [1]. The tumor's presentation can vary widely, ranging from asymptomatic cases discovered incidentally during imaging studies to symptomatic presentations with respiratory distress or systemic symptoms due to associated paraneoplastic syndromes.

The spectrum of thymoma pathology ranges from benign to malignant, and their diverse clinical presentations can complicate diagnosis and management [3]. While imaging techniques such as CT and MRI have improved diagnostic capabilities, definitive diagnosis often necessitates histological examination. Thymomas are classified based on histological features, with the World Health Organization (WHO) system categorizing them into several types from A to C [4]. This classification is pivotal for guiding treatment strategies and prognosis. Management of thymoma primarily involves surgical resection, which remains the cornerstone of treatment [5]. However, the approach may be complicated by tumor stage, histological subtype, and the presence of comorbidities. Thymoma is a very uncommon neoplasm of the thymus and has a good prognosis as compared to its malignant counterpart.

The following report is of two cases having similar presentations. In the cases in the study, a 41-year-old male and a 39-year-old female both presented with a mediastinal mass with accompanying symptoms of MG. This report delves into the histological types of thymoma, showcasing the types seen in the cases under study and the follow-up period after surgery, with an added note on the pathogenesis of MG associated with thymoma.

## Case presentation

Case 1

**Figure 1:**
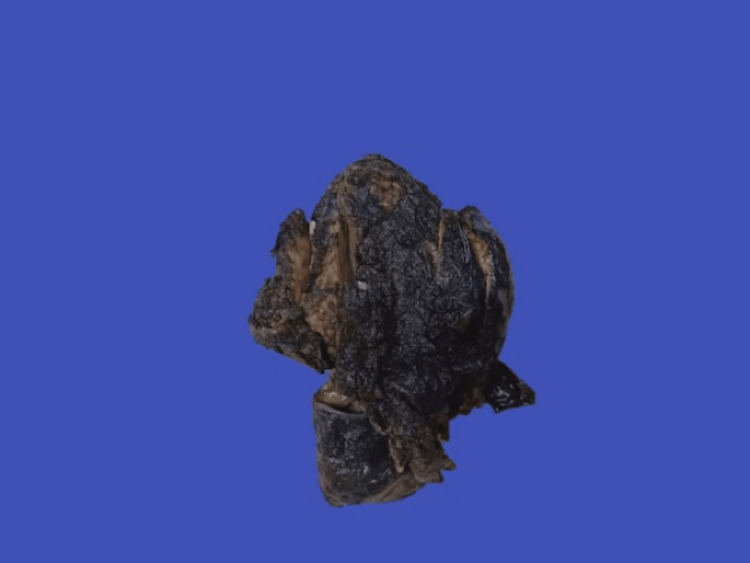
Gross image of the thymic mass showing an intact capsule

**Figure 2:**
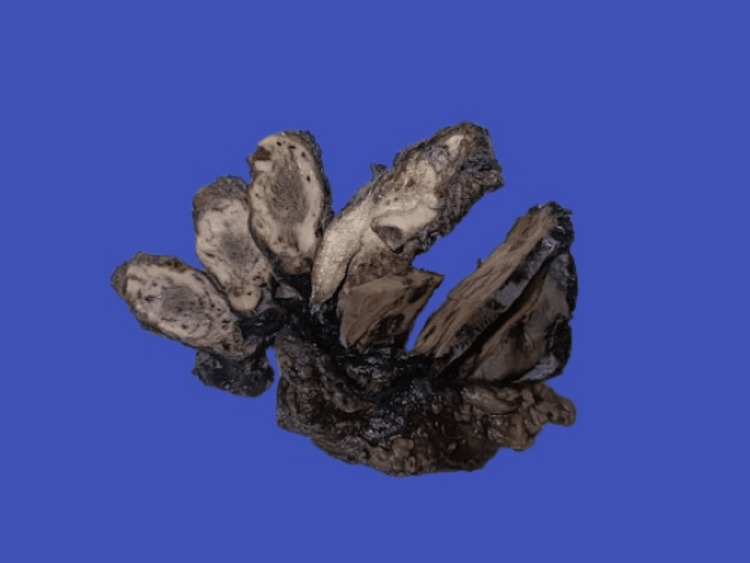
Serial sections of the mass showing grey white with central dark grey areas

The first case is that of a 41-year-old male, with a known case of MG, which was diagnosed when he was in his early 20s. He came with complaints of breathlessness with difficulty in swallowing for two months with weight loss. Radiological investigations showed an anterior mediastinal mass, and the patient underwent sternotomy with complete excision of the mediastinal mass, and it was sent for histopathological examination. On macroscopic examination, the mass was encapsulated and measured at 7.6x3.2x2.1 cm. There was no capsular breech, and the external surface was unremarkable. On serial sectioning of the mass, grey-white areas with central dark grey regions were noted (Figures 1-2).

**Figure 3:**
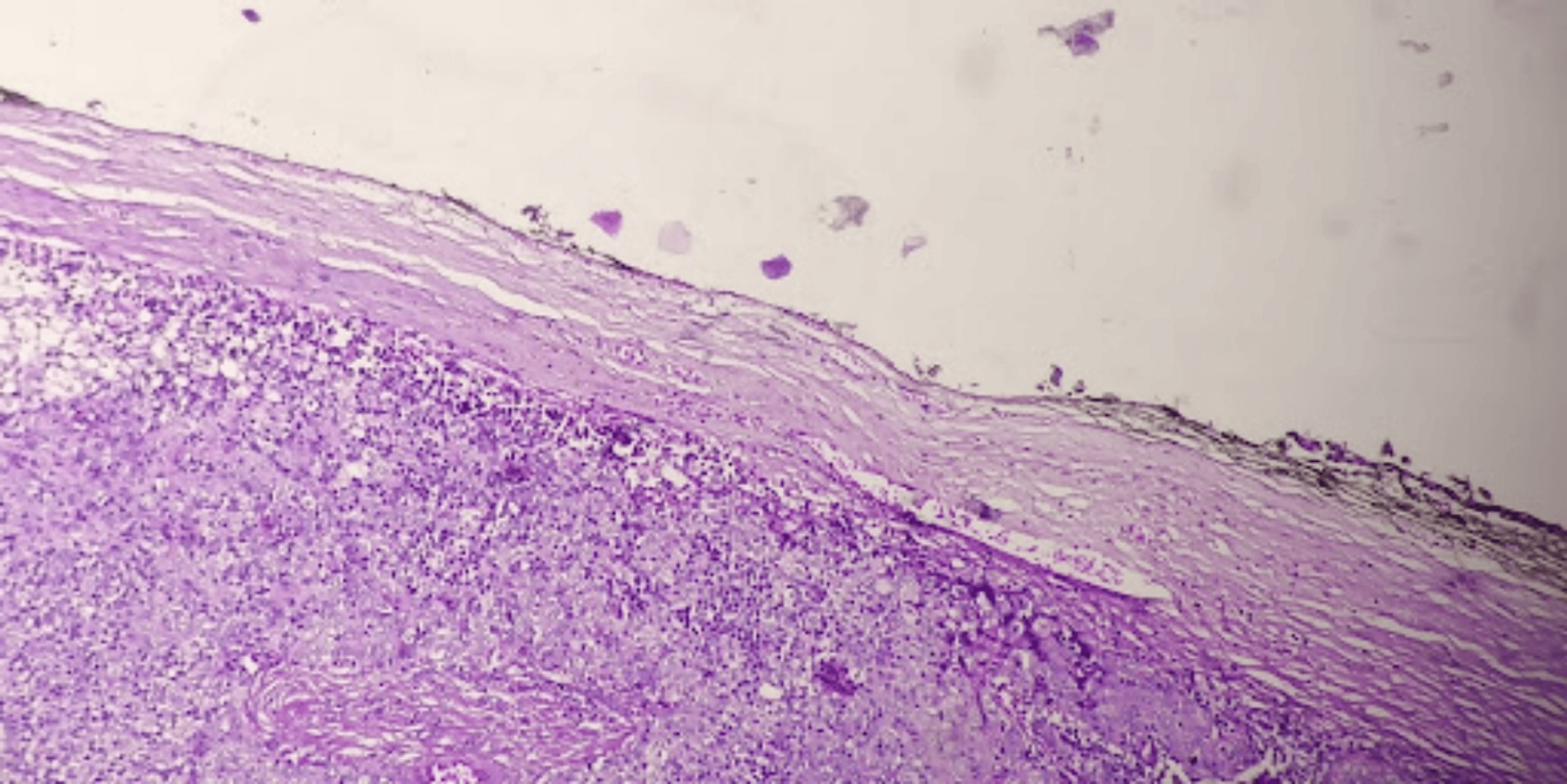
Well-encapsulated neoplasm showing an intact capsule Hematoxylin and eosin stain (20x objective)

**Figure 4:**
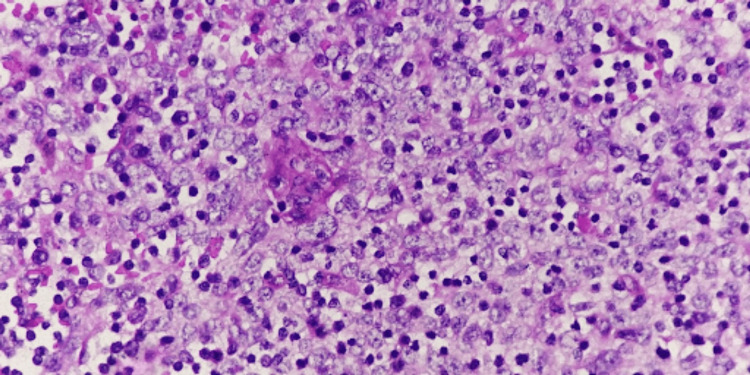
Neoplasm composed of plump epithelial cells mixed with normal thymocytes Hematoxylin and eosin stain (40x objective)

Microscopic examination of the sections submitted from the mass showed a well-encapsulated neoplasm with no capsular invasion. The neoplasm was composed of plump epithelial cells arranged in sheets mixed with normal thymocytes (Figures 3-4).

There was no associated pleural, lung, or pericardial involvement, and no lymph nodes were submitted for examination. Based on the above microscopic examination, this tumor was assigned a histologic type of B2.

Case 2

**Figure 5:**
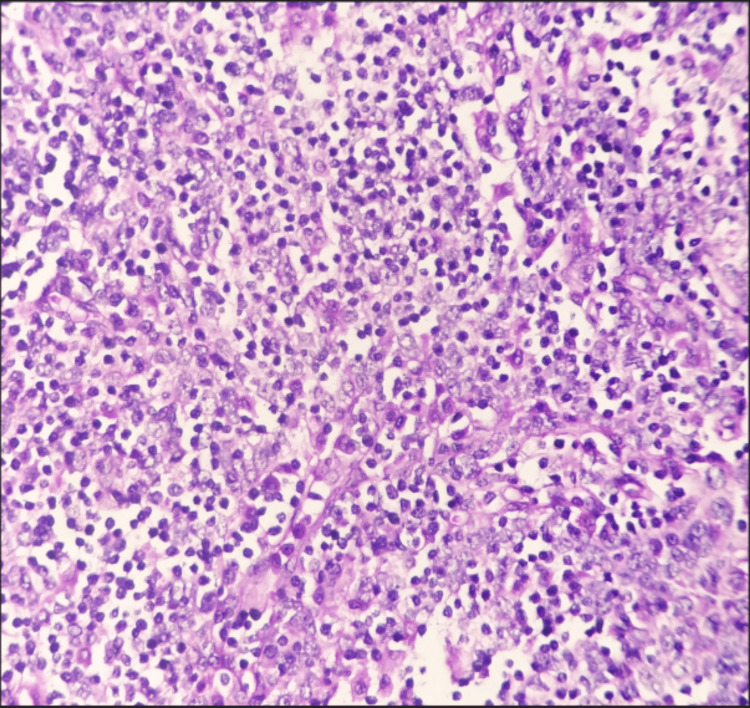
Neoplasm composed of fewer epithelial cells as compared to normal thymocytes Hematoxylin and eosin stain (40x objective)

The second case is that of a 39-year-old female who came with complaints of difficulty in swallowing solids for the past three months, with a history of double vision and drooping of eyelids. Radiological investigations showed a similar anterior mediastinal mass, as seen in the previous, which was most likely a thymic enlargement. Subsequently, the patient underwent excision of the mass, which was sent for histopathological examination. Upon microscopic examination, very few neoplastic plump epithelial cells were seen compared to the normal thymocytes. There was no lymphovascular invasion, and no lymph nodes were seen or submitted separately. Thus, in view of the above findings, this mass was found to be a thymoma of histologic type B1 (Figure 5).

Following complete resection of the masses, the post-operative period was uneventful, and both of the cases in our study showed significant improvement in the myasthenia symptoms. On follow-up, which was done every six months for a period of one and a half years, there was no documentation of any recurrence of the mass or reemergence of the previous symptoms.

## Discussion

Originating from the thymus's epithelial cells, thymoma is an uncommon type of tumor. They have an incidence of 0.13-0.26 cases per 100,000 population [1]. It is frequently associated with a wide range of paraneoplastic syndromes and other autoimmune conditions, out of which MG is the most common. A study by Mao et al. showed that male MG patients had a higher risk of developing thymoma [6]. Grossly, it appears as a well-circumscribed, lobulated mass with a tan-white cut surface with a lobulated architecture [7]. Microscopically, it is classified based on the cytological features present. Thymic neoplastic epithelial cells can be either bland and spindled or polygonal and plump. Based on this cytomorphology, thymomas are classified into type A (spindle), type B (plump), and type AB (mixed). Type B is further subclassified into three types, based on the ratio of tumor cells to thymocytes, with B1 having the least tumor cells and B3 having more tumor cells than thymocytes [8]. It is staged on the basis of the Masaoka-Koga system [9,10].

Thymoma is associated with a wide range of paraneoplastic syndromes, including MG, Lambert-Eaton syndrome, myositis, encephalitis, acquired neuromyotonia, Morvan’s syndrome, and pure red cell aplasia [11]. Among these, MG is the most common, with 15-20% of myasthenia patients having a thymoma and about 30% of thymoma patients developing MG [12]. Kondo et al. conducted a study revealing that approximately 10% of patients with myasthenia also have thymoma, while approximately 33% of patients with thymoma also have myasthenia instead [13].

MG is an autoimmune disorder caused by antibodies against acetylcholine receptors (AchR) [14,15]. MG clinically manifests as symptoms of muscle weakness, such as weakness in the arms, hands, fingers, legs, and neck; drooping of one or both eyelids (ptosis); blurred or double vision (diplopia); and difficulty in swallowing [16]. Based on the type of antibody involved, MG is classified into antibodies against AChR, against muscle-specific kinase (MusK), and against agrin receptor LRP4 [17].

The thymus or thymoma plays a crucial role in the pathogenesis of AchR MG. After an unknown trigger, thymic epithelial cells of the major histocompatibility complex (MHC) II class present unfolded AChR subunits and activate auto-reactive CD4+ T lymphocytes. The antibodies from the T cells in turn activate complement and lead to the formation of AChR-immune complexes, leading to further activation of B cells and subsequent production of AChR antibodies [18]. Though according to earlier literature, the presence of MG in thymoma patients is an indicator of poor prognosis [19], recent studies showed that MG no longer influences the prognosis of thymoma [20-23]. In regards to the cases in this study, both of them following surgery showed significant improvement of the MG symptoms, and recent follow-ups showed no signs of any recurrence.

## Conclusions

Thymoma is an epithelial neoplasm of the thymus associated with a wide range of paraneoplastic syndromes. Among them, MG is the most commonly associated immune condition. In conclusion, this case report highlights the complexities involved in the diagnosis of thymoma. Both of our patients presented with symptoms of the associated autoimmune conditions, emphasizing the importance of a thorough clinical evaluation. Surgical resection proved to be the cornerstone of treatment in both cases. This also emphasizes the need for a multidisciplinary approach in managing thymoma, involving thorough clinical evaluation, imaging modalities, and histopathological examination. Continued exploration of thymoma's pathophysiology and its relation to other paraneoplastic syndromes and treatment options will be essential for improving the prognosis and quality of life of affected patients.
